# Behavioral Patterns of Care Providers and Resistiveness to Care of Persons With Dementia

**DOI:** 10.1093/geroni/igaa057.519

**Published:** 2020-12-16

**Authors:** Bo Ra Kim, Kyung Hee Lee, Eunhee Cho, Heejung Kim, Bora Kim

**Affiliations:** 1 Yonsei University, Seoul, Republic of Korea; 2 Yonsei University College of Nursing, Seoul, Republic of Korea

## Abstract

Resistiveness to care in persons with dementia is a distinct obstacle for care providers to provide oral care. It is necessary to find out specific behaviors of care providers that affect resistiveness to care. This is a secondary data analysis to identify behaviors of care providers related to resistiveness to care using 70 videos that were taken when 23 persons with dementia received oral care in long-term care facilities at baseline, month 3 and month 6. The behavior patterns of care providers and resistiveness to care of persons with dementia were extracted in the form of the frequency of each behavior using a coding scheme for behavioral observation. The coding scheme for behavior patterns of care providers consists of 2 dimensions: person-centered behaviors including 18 items and task-centered behaviors including 4 items. In addition, the coding scheme for resistiveness to care of persons with dementia includes 13 items. The data were analyzed using a linear mixed model analysis considering the repeated measured data. Among person-centered behaviors, ‘assessing the comfort of resident,’ and ‘cooperatively negotiating’ were less likely to occur resistiveness to care. However, ‘physically controlling’ was the most significant to increase the occurrence of resistiveness to care of persons with dementia. Therefore, it is recommended that care providers try to understand the causes of discomfort for persons with dementia and meet his/her needs rather than to physically control when providing oral care.

